# Impact of inadequate empirical antibiotic treatment on outcome of non-critically ill children with bacterial infections

**DOI:** 10.1186/s12887-024-04793-0

**Published:** 2024-05-11

**Authors:** Amit Dar, Tali Bdolah Abram, Orli Megged

**Affiliations:** 1 Department of Internal Medicine, Hadassah-Hebrew University Medical Center, Ein Kerem, Jerusalem, Israel; 2 Faculty of Medicine, Hebrew University of Jerusalem, Jerusalem, Israel; 3 The Pediatric department and pediatric infectious diseases unit, Shaare Zedek Medical Center, P.O.B. 3235, Jerusalem, Israel

**Keywords:** Inadequate antibiotic treatment, Children, Risk factors, Bacteremia, Renal abnormalities

## Abstract

**Background:**

The impact of inadequate empirical antibiotic treatment on patient outcomes and hospitalization duration for non-life-threatening infections in children remains poorly understood. We aimed to assess the effects of inadequate empirical antibiotic treatment on these factors in pediatric patients.

**Methods:**

The medical records of children admitted for infectious diseases with bacteria isolated from sterile sites between 2018 and 2020 were retrospectively reviewed. Patients who received adequate empirical treatment were compared with those who received inadequate treatment in terms of demographic, clinical, and laboratory variables.

**Results:**

Forty-eight patients who received inadequate empirical antimicrobial treatment were compared to 143 patients who received adequate empirical treatment. Inadequate empirical antimicrobial treatment did not significantly affect the length of hospital stay or the incidence of complications in non-critically ill children with bacterial infections. Younger age and underlying renal abnormalities were identified as risk factors for inadequate antimicrobial treatment, while associated bacteremia was more common in the adequate antimicrobial treatment group.

**Conclusions:**

inadequate antibiotic treatment did not affect the outcomes of non-critically ill children with bacterial infectious diseases. Therefore, routine empirical broad-spectrum treatment may not be necessary for these cases, as it can lead to additional costs and contribute to antibiotic resistance. Larger prospective studies are needed to confirm these findings.

## Background

Empiric antimicrobial therapy is the initial choice of antimicrobials pending microbiological results, and should cover the pathogens that are likely to cause the infection, taking into account the patient’s age, the site of infection, medical history and other factors. Adequate antibiotic use is commonly defined as the use of an antimicrobial agent that is correct based on all available clinical, pharmacological, and microbiological data. Broad-spectrum therapy is recommended for initial empiric therapy of critically ill children with sepsis [1]. There are guidelines for the empirical antibiotic treatment in several common pediatric situations, including the management of young infants < 60 days with fever [2], management of fever without source in infants and children < 3 years before [3] and after [4] the engagement of the conjugated pneumococcal vaccine into the routine immunization program, and management of urinary tract infections in children < 24 months [5]. However, selecting adequate empirical antibiotic therapy for children with suspected bacterial infections in other circumstances can be challenging and requires a number of considerations, including past medical history and comorbidities, site of infection and local antibiotic susceptibility patterns.

Inadequate therapy can influence patient outcomes, while broad-spectrum treatment can lead to antibiotic resistance and can influence gut microbiome [6]. One of the consequences of the rise in antibiotic resistance is the growing rate of inadequate empirical antimicrobial treatment of bacterial infections. Adequate empirical antibiotic therapy improves the outcomes for patients with severe sepsis, pediatric patients with severe pneumonia [7], children with nosocomial bloodstream infection [8], neonates with gram positive and gram negative bacteremia [9, 10] and more. However, the significance of adequate empirical antibiotic treatment in children with milder infections is debatable [11, 12] and may be influenced by many factors, such as infection severity, the source of infection, the patient’s immune status, and the pathogenicity of the causative organism. Little is known about the implications of inadequate empirical antibiotic treatment of bacterial infections in not critically ill children.

## Materials and methods

This study was a retrospective analysis of pediatric patients 0–18 years old with microbiologically confirmed bacterial infections, with bacteria isolated from sterile sites, who were admitted to a university-affiliated general hospital between 2018 and 2020. The sample size was calculated according to the results of a small pilot study showing 1.5 days longer hospitalization in the group of patients with inadequate empirical treatment. Using power of 80% and 5% α type mistake (α -0.05), and defining 1:3 ratio between the inadequate and adequate treatment groups, the calculated sample size was 48 and 144 patients in each group. The patients were identified through screening of the computerized microbiological database, and then review of the medical records of patients with positive cultures. Preterm infants were included if their post-conception age was > 40 weeks. The following data were retrieved from the medical records: patient age and sex, ethnicity, underlying conditions and co-morbidities, previous admissions, date of admission, duration of illness before admission, temperature at admission, complete blood count (CBC), C-reactive protein (CRP), causative bacteria, presence of bacteremia, empirical antimicrobial treatment, adequacy of empirical antimicrobial treatment, length of hospitalization, discharge diagnosis, infectious complications and outcome. Children who were admitted to the pediatric intensive care unit (PICU) or neonatal intensive care unit were excluded from the study. All patients that were included in the study were hemodynamically and neurologically stable.

Urinary tract infection was defined as pyuria and > 50,000 CFU/ml of a single urinary pathogen. Gastrointestinal infection was defined as having more than 3 watery stools in 24 h. Antibiotics were empirically used for gastroenteritis in cases of bloody diarrhea and for gastroenteritis with prolonged fever where *Salmonella* bacteremia was clinically suspected. Other gastrointestinal illnesses included perforated appendicitis and ascending cholangitis (in patients with biliary atresia who underwent Kasai procedure).

Head and neck infections included mastoiditis, otitis media with associated bacteremia and bacterial parotitis. Pneumonia was defined as combination of clinical signs and symptoms (such as fever and cough) with radiologic documentation of lobar infiltrates.Hospital acquired infection was considered if signs and symptoms started more than 48 h after admission to the hospital.

We defined Inadequate antibiotic therapy as the microbiological documentation of an infection with a causative pathogen that was resistant to the empirical treatment based on in-vitro susceptibly tests. When multiple antibiotic treatments were prescribed, we defined inadequate therapy is the pathogen was resistant to all empirical antibiotics prescribed. Antibiotic penetration to the site of infection and adequate dosing were also taken into consideration by a pediatric infectious diseases specialist. The following pathogens in blood cultures were categorized as contamination and were excluded: coagulase-negative staphylococci, *Corynebacterium* species, *Bacillus* other than *Bacillus anthracis*, *Cutibacterium acnes*, alpha-hemolytic streptococci other than *Streptococcus pneumoniae*, *Acinetobacter* species other than *Acinetobacter baumannii*, *Micrococcus* species and nonfermenting Gram-negative organisms, including *Pseudomonas* other than *Pseudomonas aeruginosa* and *Neisseria* other than *Neisseria meningitidis*.

Patients who received adequate empirical treatment were compared with those who received inadequate treatment in terms of demographic, clinical, and laboratory variables.

The primary endpoint was the length of hospitalization in days, from emergency department admission to discharge. The secondary endpoints included time to switch to oral antibiotic treatment (some patients were discharged with intravenous home therapy) and the presence of long-term complications as documented in the discharge letter or ambulatory follow-up (need for rehabilitation in a long term facility, neurological deficits at discharge after meningitis)Antimicrobial susceptibility testing was performed using the disk diffusion method, according to the CLSI standards [13]. ESBL determination was performed phenotypically with ceftazidime/ceftazidime clavulanate and cefotaxime/cefotaxime clavulanate disks, as recommended by the Clinical and Laboratory Standards Institute (CLSI) [13].

### Statistical analysis

Data was collected using Microsoft Excel 2010 sheet. Statistical analysis was done using IBM SPSS Software version 25. Kolmogorov-Smirnov test was used for continuous variables to evaluate for normal distribution. Qualitative variables were compared by chi-square test. When the number of expected frequencies was low, the variables were compared using Fisher’s exact test. Quantitative variables were compared by the t-test. ANOVA (for Quantitative variables) and Logistic regression (for dichotomic variables) were used for multivariate analysis. The variables found to be statistically significantly related to the dependent variable (length of hospital stay) in the univariate approach were introduced into a multivariate model using the ‘Stepwise, Forward, Likelihood ratio’ method. All statistical tests were two-way tests and *P* value of 5% or less was considered statistically significant.

## Results

A total of 191 children were included in the study. 104 (54.5%) of the patients were females. The mean age of children was 3.5 years and the median age was 21 months. 144 (75.4%) of the children were Jewish. 31% of the patients had medical underlying conditions, including prematurity < 36 weeks (10.5%), renal anatomical or functional disorders (15.2%), immunodeficiency (3 patients with trisomy 21 and hypogammaglobulinemia, 2 patients with nephrotic syndrome and hypogammaglobulinemia, DiGeorge syndrome, IFNGR2 deficiency, renal transplantation and Wiscott Aldrich syndrome) (4.7%), cardiovascular disease (3.7%), gastrointestinal disease 2.6%)), and others (9.4%). There were no patients with cancer in this cohort. Two patients had hospital acquired infections. Two patients had central venous catheters for parenteral nutrition. 32 (17%) of the patients were treated according to Rochester criteria [14] or according to guidelines for the management of infants and children with fever without source (3, 4). 45.5% of the patients had concomitant bacteremia. The most common sources of infection were skin and soft tissue (with abscess formation and/or bacteremia), skeletal infections and urinary tract infections. The most common organisms that were isolated were *Enterobacteriales* (46%) including *Escherichia****coli***, *Klebsiella pneumoniae, Enterobacter cloacae, Citrobacter coseri and Salmonella enteritidis*, *Staphylococcus aureus* (31%) and *Streptococcus pneumoniae* (10%). Gram positive bacteria were common in skeletal and soft tissue infections (*n* = 56, 81%), while gram negative pathogens were most common in UTIs and gastrointestinal infections (67, 94%; 9, 100% respectively).

The most common antimicrobial agents that were used for empirical treatment were penicillins, cephalosporins and aminoglycosides. First generation agent was the most used among the cephalosporins, and was more commonly used in the adequate treatment group (*n* = 51 vs. *n* = 8 in the inadequate group, *p* = 0.04). There was no difference in the usage of other cephalosporin generations between the two groups of patients. Penicillins (most commonly ampicillin) and beta lactam plus beta lactam inhibitor agents were equally used in both groups as well. In non-bacteremic patients, the most common causative pathogens in UTI and abdominal infections were *Enterobacteriales*, and *S. aureus* was the most common pathogen isolated from abscesses of soft tissue infections and bone and joint infection. *Kingella kingae* was the causative pathogen in 7 cases of septic arthritis.

In the adequate empirical treatment group, Methicillin sensitive *staphylococcus aureus* (MSSA) caused 48 (81%) of the staphylococcal infections and extended spectrum beta lactamase (ESBL) producing bacteria caused 20 (43%) of the *Enterobacteriales* infections. In the inadequate empirical treatment group, all staphylococci were Methicillin resistant *staphylococcus aureus* (MRSA) and all *Enterobacteriales* were ESBL producing bacteria.

The patients’ demographic, clinical and laboratory characteristics of forty-eight patients who received inadequate empirical treatment were compared to 143 patients who received adequate empirical treatment (Table 1).

**Table 1:** patients’ demographic, clinical and laboratory characteristics

*Characteristic*	Inadequate empirical treatment (*n* = 48)	Adequate empirical treatment (*n* = 143)	Total (*n* = 191)	*P*
**Age median (IQR)**	12 (5.75–22.5)	29 (5–78)	21 (5–63)	**< 0.001**
**Gender**				**0.022**
**male n, (%)**	17, (35)	70, (49)	87, (45)	
**female n, (%)**	31, (65)	73, (51)	104, (55)	
**Ethnicity**				
**Arab n, (%)**	21 (44)	26 (18)	47 (25)	**< 0.01**
**Jew n, (%)**	27 (56)	117 (82)	144 (75)	
**Number of previous hospitalizations (median (IQR))**	0 (0–1)	0 (0–1)	0 (0–1)	0.764
**Duration of illness before admission (days) median (IQR)**	3 (1–5)	3 (1–4)	3 (1–4)	0.54
**Underlying genitourinary malformations n, (%)**	15 (31.2)	14 (9.8)	29 (15.2)	**< 0.001**
**White blood cells/µL median (IQR)**	16 (12.5–20)	13.8 (9.25–18.8)	15 (10.7–19)	0.1
**CRP mg/dL median (IQR)**	7.3 (3.2–14.7)	10 (2.93–17.1)	9.4 (3–16)	0.422
**Organism n, (%)**				
***Enterobacteriales***	30 (62.5)	58 (40.5)	88 (46.1)	**0.008**
***S. aureus***	11 (22.9)	49 (34.3)	60 (31.4)	0.143
***S. pneumoniae***	1 (2.1)	18 (12.6)	19 (9.9)	**0.048**
**Other**	8 (16.7)	22 (15.4)	30 (15.7)	0.894
**Gram negative bacteria n (%)**	34 (70.8)	72 (50.3)	106 (55.5)	0.014
**Source of infection n, (%)**				0.188
**Skin, soft tissue, skeletal**	16 (33.3)	53 (37.1)	69 (36.1)	0.664
**Urinary tract infection**	25 (52.1)	46 (32.2)	71 (37.2)	**0.014**
**Gastrointestinal**	3 (6.2)	6 (4.2)	9 (4.7)	0.843
**Pneumonia**	2 (4.2)	12 (8.4)	14 (7.3)	0.523
**Meningitis**	0 (0)	6 (4.2)	6 (3.1)	0.339
**Head and neck**	0 (0)	3 (2.1)	3 (1.6)	0.574
**Other**	1 (2.1)	4 (2.8)	5 (2.6)	1
**Bacteremia n, (%)**	11 (22.9)	76 (53.1)	87 (45.5)	**< 0.001**

Female sex, age, Arab descent, urinary tract infection (UTI) as the source of infection, underlying genitourinary abnormalities and *Enterobacteriales* were found as risk factors for inadequate empirical antimicrobial treatment, while associated bacteremia and growth of *S. pneumoniae* was associated with adequate empirical treatment. In multivariate analysis, age, lack of associated bacteremia and presence of underlying genitourinary abnormalities were found as risk factors for inadequate empirical antimicrobial treatment (Table 2).

**Table 2:** Multivariate analysis of factors associated with inappropriateness of empirical antibiotic treatment

Characteristic	OR (95% CI)	*P*
**Age**	**4.4 (1.9, 9.9)**	**< 0.001**
**Urinary tract infection** **Arab descent** **Female sex** **Bacteremia** **Underlying genitourinary malformations**	2.27 (0.96, 1.62) 2.12 (0.86, 5.17) 2.17 (0.93, 5.03) **0.46 (0.19, 0.95)** **4.22 (1.32, 13.48)**	0.419 0.063 0.119 **0.004** **0.002**
**Organism**		
***Enterobacteriales*** ***S. pneumoniae***	1.63 (0.65, 4.07) 0.34 (0.04, 2.66)	0.657 0.265

The primary endpoint was hospitalization length. There was no difference in this parameter between the groups of adequate and inadequate empirical therapy, with median length of hospitalization being 7 days for both groups. The secondary endpoints were time until switch to oral antibiotic treatment and the presence of long-term complications. Time until switch to oral treatment was shorter for children treated empirically with inadequate treatment. There were only 4 children with long term complications, all in the group of adequate empirical treatment, with no statistically significant difference between the two groups of patients. There was no difference in any of the outcome measures in sub analysis by source of infection. Number of patients with long term complications was too small for sub analysis by source of infection. The comparison of outcome measures between the two groups is presented on Table 3.

**Table 3:** Patients’ outcome according to appropriateness of empirical treatment

Characteristic	Inappropriate empirical treatment (*n* = 48)	Appropriate empirical treatment (*n* = 143)	Total (*n* = 191)	*P*
**Length of hospitalization (days) median (IQR)** **Skin, soft tissue, skeletal** **Urinary tract infection** **Bacteremia**	7 (4–10) 6 (3–12) 7 (4–9) 8 (6.5–13)	7 (4–12) 8 (4–12) 5(4–7) 9.5 (6–13)	7 (4-10.5) 7 (4–12) 5 (4–8) 9 (6-13.5)	0.332 0.528 0.238 0.83
**Days until PO antibiotics (mean ± SD)** **Skin, soft tissue, skeletal** **Urinary tract infection** **Gastrointestinal** **Pneumonia** **Bacteremia**	7.1 ± 6.23 11.52 ± 13.57 4.93 ± 3.09 11 ± 5.29 4 ± 1.41 16 ± 6.37	10.63 ± 11.29 14.32 ± 12.81 6.2 ± 3.52 8.66 ± 7.81 6.17 ± 3.69 15 ± 8.74	9.74 ± 10.36 13.3 ± 13.1 5.91 ± 3.45 9.44 ± 6.82 5.86 ± 3.51 13.8 ± 13.2	**0.008** 0.696 0.121 0.615 0.209 0.629
**Long term complications n (%)**	0 (0)	4 (2.8)	4 (2.1)	0.574

## Discussion

In this study we found that there was no difference in the outcomes of non-critically ill children admitted due to bacterial infections according to the adequateness of the empirical antimicrobial therapy. We also identified risk factors that were associated with inadequate empirical therapy, including younger age and underlying genitourinary abnormalities, whereas bacteremia was associated with adequate empirical therapy.

Young age and underlying genitourinary abnormalities are known predisposing factors for UTIs caused by resistant organisms [15], and can thus explain the high rate of inadequate empirical therapy in these cases. In our hospital, UTI is typically treated empirically with 2nd generation cephalosporins which are inadequate for extended spectrum beta lactamases (ESBL) producing bacteria and for pseudomonas infections, or with ceftazidime (for children with severe underlying malformations or decreased renal function) which is also ineffective for infections caused by ESBL bacteria. In this study, bacteremia was associated with receiving adequate empirical therapy, probably because the majority of patients with bacteremia in our cohort had penicillin sensitive pneumococcal bacteremia (19% of bacteremic cases), methicillin sensitive *S. aureus* (33%), or *S. pyogenes* and *N. meningitidis* (7%) which are always sensitive the the empirical treatment given for the diagnoses for which these patients were admitted.

Inadequate empirical treatment was not linked to worse outcome measures. These results concur with some [12, 16] but not all studies [17, 18]. The conflicting findings in the literature may result from variations in many factors, such as study populations, infection severity, the source of infection, the patients’ immune status and the pathogenicity of the causative organism.

Our study’s population is unique, as to the best of our knowledge it is the only one that looked into the effects of inadequate empirical treatment in children with non-life-threatening bacterial infections. Because early adequate empirical antibiotic treatment in life-threatening infections is known to be related with lower fatality rates and improved outcomes for both adults [10, 19–22] and children [23, 24], we did not include children who were hospitalized to the PICU.

The severity and prognosis of the disease may be influenced by the pathogenicity of the causative organism and the site of infection. Gram positive infections were typically associated with skin, soft tissue, skeletal or pulmonary infections, whereas gram negative infections typically originated from the gastrointestinal tract or UTI. Although gram negative bacteremia was more common in the inadequate empirical treatment group, the length of stay of adequate vs. inadequate empirical treatment for infections caused on by gram-positive organisms, gram-negative organisms, and various infection sites did not differ. Conflicting information can be found in the literature on the significance of the causative organisms and the infection site. Adult Methicillin-resistant *Staphylococcus aureus* (MRSA) bacteremia was not linked to greater mortality rates in patients getting insufficient empirical medication, according to two sizable studies [25, 26]. On the other hand, Lodise et al. found that delayed therapy for nosocomial *Staphylococcus aureus* bacteremia increased death and lengthened hospital stay [27]. The difference between these studies might be due to different populations, with hospital acquired bacteremia occurring in patients with comorbidities and higher risk for complications comparing to community acquired bacteremia. Regarding pneumococcal bacteremia, the majority of studies found a higher mortality risk in patients who received insufficient empirical therapy [28, 29]. However, these studies included only adult patients with mortality rates between 20 and 50%, including mortality within 24 h after admission, so the role of inadequate treatment in the outcome is difficult to assess and certainly cannot be extrapolated to the pediatric population in which mortality rates from pneumococcal bacteremia are extremely low, and bacteremia often resolves spontaneously or with oral antibiotic treatment [30]. As for gram negative bacteremia, some studies found an association between inadequate empirical antimicrobial therapy and increased mortality [31, 32], whilst other studies did not [23]. UTI is often the source of gram negative pediatric infections. A recent meta-analysis that looked at the effect of adequate versus inadequate empirical antibiotic therapy on mortality found that although rates of mortality were significantly in favor of adequate therapy, specifically for UTIs there was no statistically significant difference between the groups [33].

Our study has a few potential limitations. Because of its retrospective nature, some of the data were not accessible for all study patients, thus limiting the power of the analysis for those variables. Second, it was conducted in a single medical center and thus may reflect the local demographics and antibiotic susceptibility patterns, which does not necessarily apply to other populations. Possibly, the two groups differed due to confounders not examined in this study, which might have influenced the patients’ outcomes. The relatively small sample size may limit the power of the study to recognize other risk factors for inadequate empirical therapy; consequently, larger prospective studies are required to address this issue. Also, this study included different sources of infection and different pathogens.

## Conclusion

Despite these limitations, there was no difference between the groups receiving adequate and inadequate empirical antibiotic treatment in terms of any of the outcome criteria. We believe that for children with non-life-threatening infections, there is no routine need for empirical broad-spectrum antibiotic treatment, as inadequate empirical treatment does not pose additional risk for the pediatric patients, but can contribute to the development of antibiotic resistance and adverse drug events.

## Data Availability

The original contributions presented in the study are included in the article, further inquiries can be directed to the corresponding author.

## Abbreviations

CBC: complete blood count
CRP: C-reactive protein
ESBL: extended spectrum beta lactamase
MRSA: Methicillin resistant *staphylococcus aureus*
MSSA: Methicillin sensitive *staphylococcus aureus*
PICU: pediatric intensive care unit
UTI: urinary tract infection
